# B10 Cell Frequencies and Suppressive Capacity in Myasthenia Gravis Are Associated with Disease Severity

**DOI:** 10.3389/fneur.2017.00034

**Published:** 2017-02-10

**Authors:** John S. Yi, Melissa A. Russo, Janice M. Massey, Vern Juel, Lisa D. Hobson-Webb, Karissa Gable, Shruti M. Raja, Kristina Balderson, Kent J. Weinhold, Jeffrey T. Guptill

**Affiliations:** ^1^Division of Surgical Sciences, Department of Surgery, Duke University Medical Center, Durham, NC, USA; ^2^Department of Neurology, Neuromuscular Section, Duke University Medical Center, Durham, NC, USA

**Keywords:** myasthenia gravis, AChR, B10, regulatory B cells, Breg, IL-10

## Abstract

Myasthenia gravis (MG) is a T cell-dependent, B cell-mediated disease. The mechanisms for loss of self-tolerance in this disease are not well understood, and recently described regulatory B cell (Breg) subsets have not been thoroughly investigated. B10 cells are a subset of Bregs identified by the production of the immunosuppressive cytokine, interleukin-10 (IL-10). B10 cells are known to strongly inhibit B- and T-cell inflammatory responses in animal models and are implicated in human autoimmunity. In this study, we examined quantitative and qualitative aspects of B10 cells in acetylcholine receptor autoantibody positive MG (AChR-MG) patients and healthy controls. We observed reduced B10 cell frequencies in AChR-MG patients, which inversely correlated with disease severity. Disease severity also affected the function of B10 cells, as B10 cells in the moderate/severe group of MG patients were less effective in suppressing CD4 T-cell proliferation. These results suggest that B10 cell frequencies may be a useful biomarker of disease severity, and therapeutics designed to restore B10 cell frequencies could hold promise as a treatment for this disease through restoration of self-tolerance.

## Introduction

Autoimmune myasthenia gravis (MG) is regarded as a T cell-dependent, B cell-mediated disease. The immunopathologic mechanisms that perpetuate the disease are not fully defined, but loss of self-tolerance in the thymus is likely an early event (1). Prior studies demonstrated impaired function of FOXP3 + regulatory T cells (Tregs) in experimental models of MG and patients with established MG and acetylcholine receptor autoantibodies (2–4). However, Treg numbers were not altered in patients with muscle-specific kinase (MuSK) autoantibodies, and CD39 expression, a proposed marker of Treg functional capacity, was similarly preserved (5).

Investigations of other immune regulatory mechanisms in the disease are scant, but initial evidence in murine studies suggest that B cells with regulatory function play an important role in promoting immune tolerance (6–12). In humans, lower frequencies or defective regulatory B cells (Bregs) have been associated with lupus, multiple sclerosis, MG, and transplantation (13–19). These studies also demonstrate the difficulty of studying Bregs because the phenotype used to identify Bregs varied between investigative groups. Currently, a known specific transcription factor or cell surface phenotype that truly identifies Bregs is unknown. In addition, it is difficult to detect Bregs based on their low frequencies in circulation. Several subsets of B cells have been proposed to contain a Breg population including CD24^hi^CD38^hi^, CD25^hi^, and CD1d^hi^CD5^+^ B cells. However, the most accepted measurement of Bregs’ suppressive function is through the production of interleukin-10 (IL-10) (6, 20, 21).

A subset of Bregs with known immunosuppressive function is B10 cells. B10 cells are defined as B cells capable of producing IL-10 for eliciting suppressive function (22). This rare population normally constitutes about 0.6% of peripheral B cells in humans and has been shown to strongly regulate inflammatory T cell responses in animal models of several autoimmune diseases (10, 22, 23). Due to their low frequency in the periphery, detection of B10 cells requires a short-term stimulation with CD40L and lipopolysaccharide (LPS) or CpG along with restimulation with phorbal 12-myristate 13-acetate (PMA) and ionomycin (23). This procedure allows detection of IL-10 in B10 cells and in B10 progenitor cells. In MG, a prior study demonstrated reduced frequencies of B10 cells in patients with anti-MuSK antibodies (18). In addition, MG patients who are responders to the B cell-depleting monoclonal antibody, rituximab, have early repopulation of B10 cells following treatment (17). These initial findings support further investigation of the role of B10 cells in MG.

In this study, our goal was to further define the role of B10 cell populations in AChR MG patients by determining the capacity to generate IL-10-producing B cells, the conditions required to do so, as well defining the suppressive capacity of B10 cells from patients with MG on T cell activation. To accomplish our goals, we optimized the assay for detection of B10 cells and determined B10 cell frequencies in control and MG patients. We also show that B10 cell production is accompanied by mRNA transcription of IL-10 followed by detection of secreted IL-10. Finally, we demonstrate that B10 cell suppressive capacity on CD4 T cell proliferation in AChR MG patients is associated with disease severity and suggest that B10 cell suppression of T cells does not require cell-to-cell contact.

## Materials and Methods

### Study Population and Controls

Sixty-four MG patients were enrolled from the Duke MG clinic. All patients had detectable anti-AChR binding antibodies according to commercially available testing (Mayo Medical Laboratory, Rochester, MN, USA) as well as clinical and electrodiagnostic features consistent with the disease. Key clinical variables, including demographics, thymectomy status, and immunosuppressive medications, were recorded (Table S1 in Supplementary Material). Disease severity was defined according to the MGFA Severity Class and MG-Manual Muscle Test (24, 25). Blood samples were obtained from 21 healthy controls, who were age and gender matched to the patients as closely as possible, weighed more than 110 pounds, and were not receiving therapy for any chronic disease. Collection of samples was approved by the Duke University Institutional Review Board and written and informed consent was obtained from each patient and normal donor.

### Isolation and Storage of Peripheral Blood Mononuclear Cells (PBMCs)

Peripheral blood was obtained by venipuncture and collected in acid-citrate-dextrose tubes (BD Vacutainer). PBMCs and plasma were separated by Ficoll density gradient centrifugation, and the PBMCs were resuspended in a 90% FBS (Gemini) and 10% DMSO (Sigma-Aldrich) freezing solution. PBMCs were viably cryopreserved and stored in vapor phase liquid nitrogen, while plasma was frozen and stored at −80°C for future use.

### Detection of IL-10-Producing B Cells

To detect IL-10-producing B cells, we utilized the protocol developed by Iwata et al. (23). Then, 2 × 10^6^ PBMCs were plated in 96-well flat bottom plates in R10 [RPMI 1640 media (Gibco) + 10% FBS + 1% penicillin–streptomycin–l-glutamine (Sigma-Aldrich)] and stimulated with LPS (10 µg/mL, Sigma-Aldrich) or CpG (10 µg/mL, Invivogen) in the presence of rCD40L (1 µg/mL, R&D Systems) for 48 h at 37°C in 5% CO_2_ incubator. For the last 5 h, cells were restimulated with PMA (1 µg/mL, Sigma-Aldrich), ionomycin (0.25 µg/mL, Sigma-Aldrich), and brefeldin A (BFA; 1 μg/mL, BDB). In some experiments, IL-21 (50 ng/mL, Peprotech) or IL-35 (50 ng/mL, Peprotech) was included in the stimulation.

After 48 h of stimulation, intracellular cytokine staining was performed to detect IL-10. First, cells were stained with CD19 PerCP-Cy5.5 (HIB19), CD27 PE (O323), IgD APC-Cy7 (IA6-2), Zombie Violet, CD14 Pacific Blue (M5E2), CD3 Pacific Blue (UCHT1), and CD16 Pacific Blue (3G8) conjugates for 25 min at 4°C. All the cell surface fluorescent antibodies were obtained from Biolegend. Following cell surface staining, cells were treated with Cytofix/Cytoperm (BDB) in accordance with the manufacturer’s recommendations. Afterward, cells were stained intracellularly with IL-10 eFluor660 (JES3-9D7, eBioscience) for 30 min at 4°C. Cells were fixed with 1% paraformaldehyde (PFA) and acquired on a LSRII flow cytometer (BDB).

### PrimeFlow Analysis of IL-10 RNA and Protein

Two million PBMCs were plated in 96-well flat bottom plates in R10 and stimulated with CpG in the presence of rCD40L for 24 or 48 h at 37°C in 5% CO_2_ incubator. Five hours before the end of these stimulations, cells were restimulated with PMA and ionomycin in the presence of BFA; unstimulated cells were also treated with BFA. The PrimeFlow assay was performed according to manufacturer’s recommendations (Affymetrix). Briefly, cells were plated in a 96-well V bottom plate and stained with CD19 PE-eFluor610 (HIB19, eBioscience), CD27 PE (O323, Biolegend), IgD APC-Cy7 (IA6-2, Biolegend), Zombie Violet (Biolegend), CD14 Pacific Blue (M5E2, Biolegend), CD3 Pacific Blue (UCHT1, Biolegend), and CD16 Pacific Blue (3G8, Biolegend) conjugates for 25 min at 4°C. Following cell surface staining, cells were treated with the kit’s fixation buffer for 30 min at 4°C followed by an RNA Perm Buffer for 30 min at 4°C. Intracellular staining was then performed for 30 min at 4°C with IL-10 PE-Cy7 (JES3-9D7, eBioscience). Cells were fixed with a second fixation buffer for 30 min at room temperature. Afterward, cells were stained with an IL-10 AlexaFluor 647 RNA probe, or a positive control RPL13A AlexaFluor 647 RNA probe, or no probe and placed in a 40°C incubator for 2 h. The next day, the probes were then amplified and labeled. Cells were resuspended in PBS and acquired on a LSRII flow cytometer (BDB).

### B10 Suppression Assay

Prior to stimulation for B10 cells, B cells were negatively isolated from a starting population of 10^7^ PBMCs, using the B cell enrichment kit (Stemcell). Isolated B cells were plated in 96-well flat bottom plates in RPMI + 10% FBS. Cells were stimulated with CpG and rCD40L for 48 h at 37°C in 5% CO_2_ incubator. At 43 h, cells were restimulated with PMA and ionomycin. In parallel with the end of the 48-h stimulation, PBMCs from the same patients were enriched for CD4 T cells (Stemcell) and stained with the Violet Proliferation dye (1 µM, BD Bioscience) in PBS and incubated in a 37°C water bath for 15 min. After 48 h, the isolated CD4 cells were combined with B cells at 1:1 and 1:2 ratio of T:B cells along with a CD4 T cell only condition and stimulated with αCD3/αCD28 for 5 days. For experiments using the transwell plates, stimulated B cells were plated in top chamber of 24-well transwell plates while CD4 T cells and αCD3/αCD28 were plated in bottom chamber.

To visualize proliferation of the CD4 T cells, cells were stained with Zombie Violet, CD14 Brilliant Violet 510 (M5E2, Biolegend), CXCR5 AlexaFluor 647 (RF8B2, BDB), CD8 AlexaFluor 700 (SK1, Biolegend), CD3 APC-Cy7 (SK7, Biolegend), CD19 PE (HIB19, Biolegend), and CD4 PE-Cy7 (SK3, Biolegend) conjugate for 25 min at 4°C. Following cell surface staining, cells were fixed with 1% PFA and acquired on a LSRII flow cytometer (BDB).

### Data Analysis

B10 cell frequencies and suppressive capacity were compared between controls and MG patients. In addition, MG patients were analyzed by disease severity according to MGFA Severity Class (mild or moderate/severe) and ocular only weakness (ocular MG) versus generalized disease. Data analysis was performed using Flowjo software (Tree Star, Ashland, OR, USA). Student’s *t*-tests were used to determine statistical significance. The *p* values were calculated using Prism software (Graph Pad, La Jolla, CA, USA).

## Results

### Detection of IL-10 RNA and Protein

**Figure 1:**
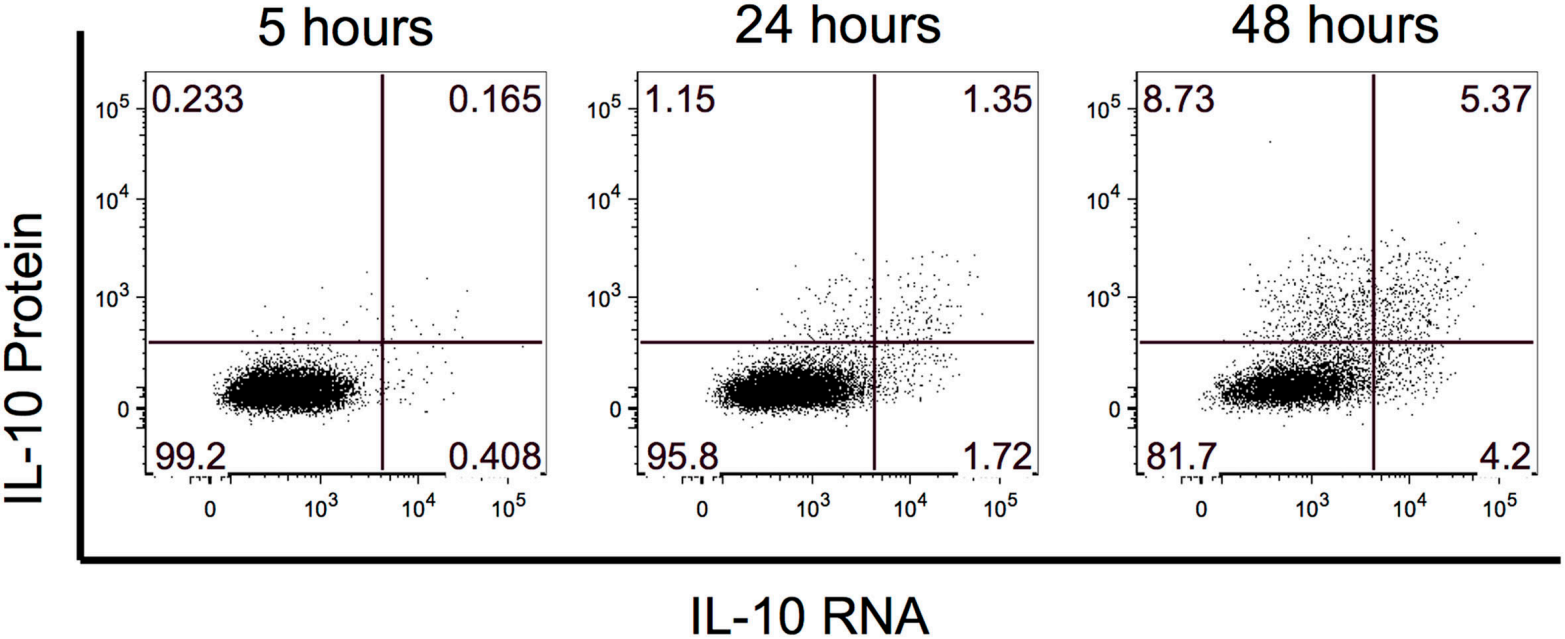
**Interleukin-10 (IL-10) expression is highest after 48 h of stimulation**. The kinetics of IL-10 RNA and protein was examined after 5, 24, and 48 h of stimulation. IL-10 RNA and protein were simultaneously detected by flow cytometry using Affymetrix’s PrimeFlow assay.

Previous B10 studies identified IL-10-producing B cells after 48 h of stimulation with LPS or CpG. To evaluate whether IL-10 RNA could be detected prior to the 48-h timepoint, we performed a PrimeFlow RNA assay to co-visualize IL-10 RNA and protein expression by flow cytometry. We examined IL-10 expression after 5, 24, and 48 h of stimulation with rCD40L and CpG, and for the last 5 h, the cells were restimulated with PMA and ionomycin along with BFA. At the 5 h timepoint, we observed hints of IL-10 RNA and protein, and this expression increased with stimulation time (Figure 1). By 48 h, we observed the highest frequency of IL-10+ events and detected three combinations of IL-10-expressing B cells including IL-10 RNA only, IL-10 protein only, and IL-10 RNA and protein. Based on the highest expression of IL-10, we focused our evaluation of B10 cells after 48 h of stimulation.

### B10 Frequency Is Associated with Disease Severity

**Figure 2:**
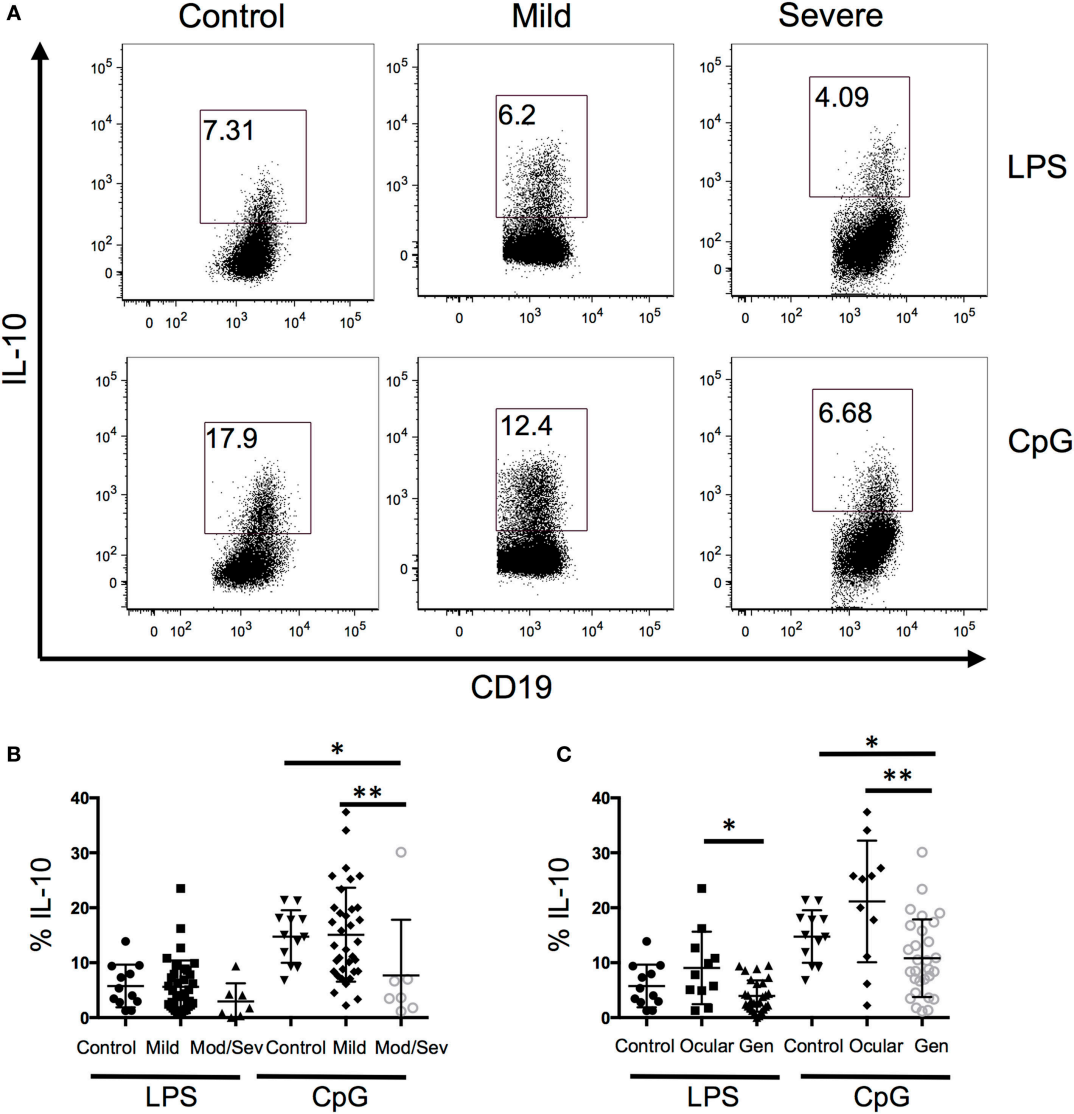
**A decrease in the frequency of B10 cells is associated with disease severity**. Intracellular cytokine staining of peripheral blood mononuclear cells after 48 h of stimulation with lipopolysaccharide (LPS) or CpG and phorbal 12-myristate 13-acetate/ION during the last 5 h. **(A)** Representative flow cytometry plots of control, mild, and severe patients. Number in the gated box represent the frequency of interleukin-10 (IL-10)+ B cells; gated on CD19^+^ cells. **(B,C)** Composite data of B10 frequencies divided by **(B)** MFGA classification (12 control, 35 mild, 7 moderate/severe) or **(C)** divided by control, ocular, or generalized disease (12 controls, 11 ocular, 28 generalized). Statistical significance is represented as follows: *p < 0.05; **p < 0.01.

Because B10 cells promote immune tolerance, we next evaluated whether the frequency of IL-10-producing B cells is associated with disease severity. When all the MG patients were grouped together and compared to controls, we did not observe a difference between the two groups; therefore, we separated the MG patients based on disease severity (Figure 2A). Disease severity of MG was categorized into mild and moderate/severe MG patients based on MGFA classifications of I–II and III–V, respectively. The lowest frequency of IL-10+ B cells was observed in the moderate/severe group and it was significantly lower compared to the control and mild groups (Figure 2B). Alternatively, we divided the MG patients into ocular only weakness and generalized disease, and the mean frequency of IL-10+ B cells in the generalized group was significantly lower than the control and ocular groups (Figure 2C). Collectively, we observed a decrease in B10 frequencies as MG severity worsened.

### Generation of B10 Cells in the Presence of IL-21 or IL-35

**Figure 3:**
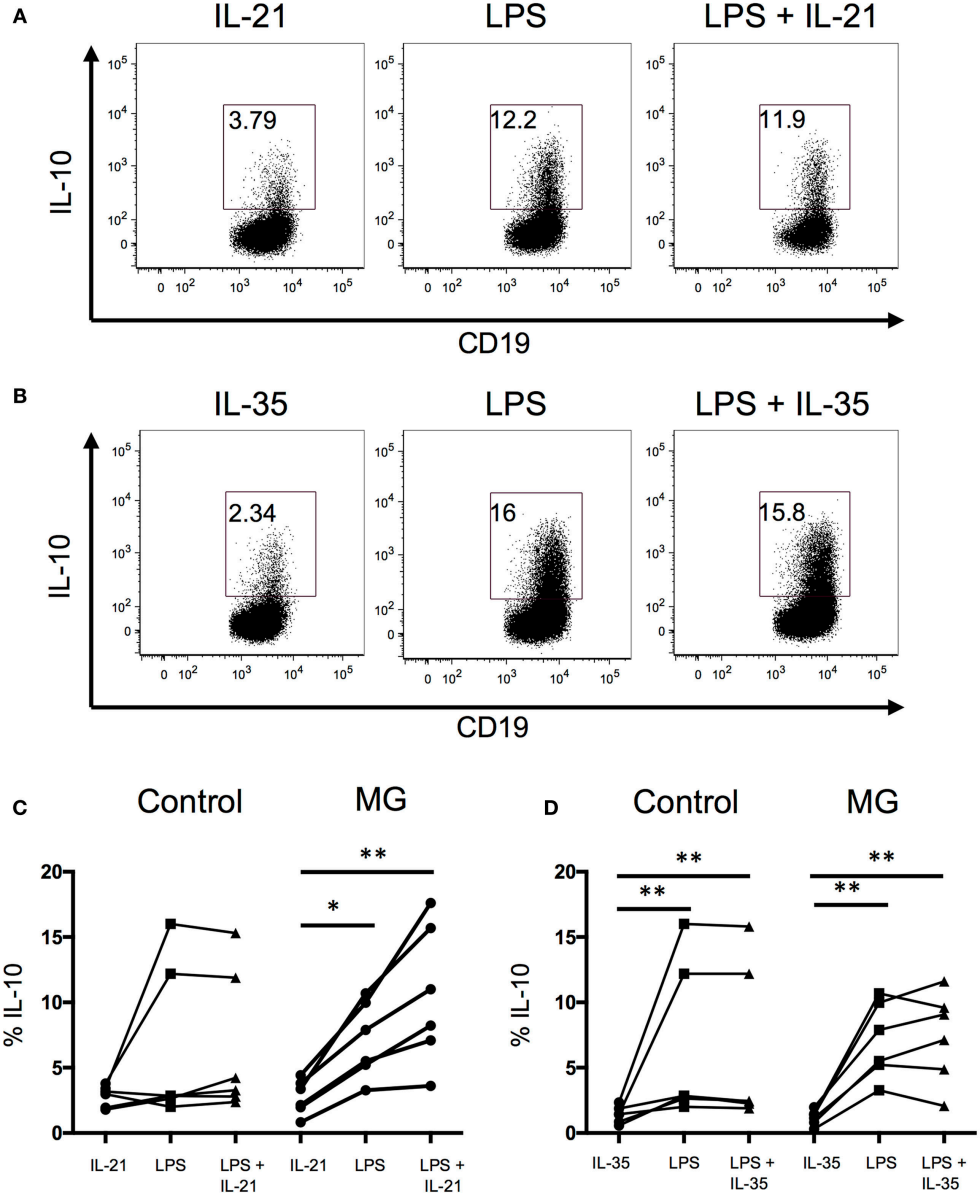
**IL-21 or IL-35 does not enhance B10 cell populations**. The effect of IL-21 or IL-35 on the generation of B10 cells was evaluated after 48 h of stimulation with lipopolysaccharide (LPS). Intracellular cytokine analysis for interleukin-10 (IL-10)+ B cells in the presence and absence of **(A)** IL-21 or **(B)** IL-35 and in combination with LPS. Bar graphs show the fraction of CD19^+^ B cells producing IL-10 after stimulations with **(C)** IL-21 or **(D)** IL-35 (six controls and six MG patients). Statistical significance is represented as follows: *p < 0.05; **p < 0.01.

Recent studies suggest that IL-21 and IL-35 are involved in the generation of B10 cells (26, 27). Thus, we examined whether the addition of IL-21 or IL-35 enhance the frequency of IL-10-producing B cells. We found that in both controls and MG patients, the addition of recombinant IL-21 or IL-35 did not enhance IL-10 production when added to the LPS or CpG stimulations (Figure 3). When cells were stimulated with IL-21 or IL-35 alone, in the presence of rCD40L, IL-21 and IL-35 induced the production of IL-10 by B cells, but the frequency of IL-10 was lower compared to toll-like receptor signaling by LPS and CpG. These results support an independent role for IL-21 and IL-35 in promoting the generation of B10 cells.

### T Cell Suppression by B10 Cells

**Figure 4:**
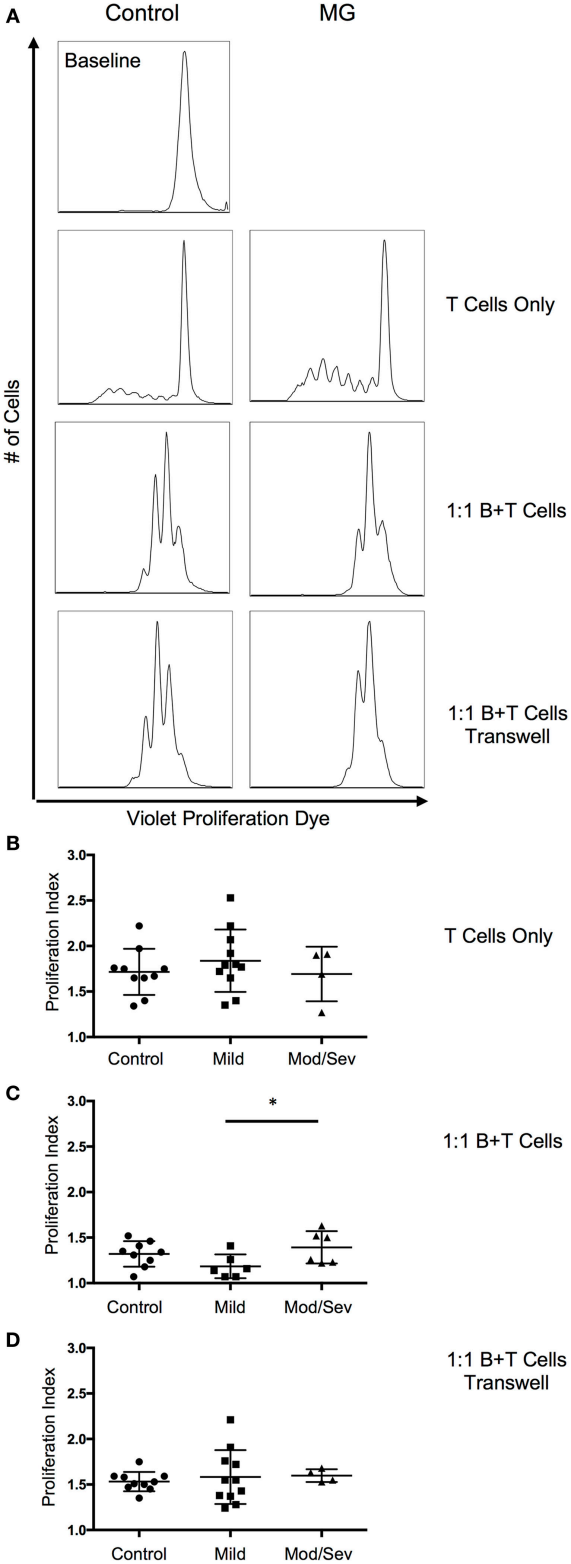
**ContinuedSuppression of T cell proliferation by B10 cells worsens with disease severity**. To evaluate suppression by B10 cells, proliferation of CD4 T cells was measured after a 5-day stimulation with anti-CD3 and anti-CD28. **(A)** Representative histograms of CD4 T cell proliferation from a control and MG patient. Baseline level represents the fluorescence of the proliferation dye prior to anti-CD3 and anti-CD28 stimulation. **(B–D)** Comparison of the proliferative index from control subjects and MG patients categorized by MGFA classifications of mild or moderate/severe. Culture conditions include **(B)** CD4 T cell only (10 controls, 11 mild, 4 moderate/severe); **(C)** 1:1 mixture of B and CD4 T cells (9 controls, 6 mild, 6 moderate/severe); and **(D)** 1:1 mixture of B and T cells in transwell plate (10 controls, 11 mild, 4 moderate/severe). Statistical significance is represented as follows: *p < 0.05.

Since we observed a decrease in B10 cells in MG patients, we next investigated the suppressive ability of B10 cells on CD4^+^ T cell proliferation. Proliferation of CD4^+^ T cells was assessed by the Violet Proliferation Dye 450, whose fluorescence diminishes by half after each division. To test the function of B10 cells, the B cells containing the newly generated subset of B10 cells after 48 h of stimulation were cultured at a 1:1 ratio of B cells and CD4 T cells in the presence of αCD3 and αCD28 stimulation. Compared to the positive control condition with only T cells, we observed a significant decrease in the proliferative index in both MG patients and controls when B cells containing B10 cells were included in the culture (Figure 4). The proliferation index is the average number of divisions underwent by those cells that divided. This decrease in the proliferative index, after the addition of B cells, was comparable between the control group and the total MG patient group. However, when MG patients were categorized by disease severity (ocular/mild or moderate/severe), the moderate to severe group were less capable of suppressing CD4 T cell proliferation compared to the mild group (Figure 4C). Furthermore, to determine if this decrease in proliferation is IL-10 dependent we introduced a transwell condition that separated the B cells and CD4 T cells from cell-to-cell contact, but allowed proteins to migrate through the permeable barrier. We observed a decrease in the proliferation of T cells in the transwell culture compared to the T cell only culture; however, this decrease was not on par with the suppression in the 1:1 coculture condition (Figure 4D).

## Discussion

Our results suggest that reduced frequencies of B10 cells are partially responsible for the loss of self-tolerance in patients with AChR antibody positive MG. The underlying immunopathogenic mechanism responsible for reduced B10 cell frequencies in MG patients is uncertain and many fundamental aspects of B10 cell biology remain to be elucidated. However, therapeutic strategies aimed at restoring B10 cell numbers may be a rational target for ameliorating MG and other autoimmune diseases with similar pathophysiology (27, 28).

A unique aspect of this study was the application of a PrimeFlow RNA assay that uses branched-DNA technology to amplify the detection of an RNA transcript in combination with staining for proteins with flurochrome-conjugated antibodies. This enabled us to assess the kinetics of IL-10 production by B cells during the assay development stage and optimize conditions (48-h stimulation with CpG) for B10 cell identification. This was particularly helpful for identifying this scarce B cell subset that is even more rare in the setting of MG. For future therapeutic strategies that seek to expand this B cell subset, this knowledge of prime B10 cell conditions derived from the PrimeFlow RNA assay will be particularly helpful.

Another potential application for the observations in our study is to monitor B10 cell frequencies as a marker of disease severity. Since B10 cell frequencies progressively decrease as disease severity increases, this cell subset could be used in the clinic to longitudinally monitor patient status in response to therapy and possibly to predict worsening. This approach appears promising in the setting of rituximab-treated MG patients where rapid repopulation of B10 cells was associated with better outcomes (17). Further validation for this application, including in the setting of other immunosuppressives, is needed.

Based on previous Breg studies demonstrating IL-21 and IL-35 as drivers of Breg development, we examined whether recombinant IL-21 or IL-35 enhances the frequency of B10 cells (26, 27). Although we found that IL-21 or IL-35 did not act synergistically with LPS or CpG to enhance the frequency of B10 cells, the addition of IL-21 and IL-35 alone promoted the generation of B10 cells. This result suggests that IL-21 and IL-35 may regulate the generation of B10 cells, and the differences between our study and the previously mentioned studies could be attributed to the disease model, animal model, and source of cells. We utilized PBMCs for our studies, while the murine studies examined splenic B cells. For future studies using IL-21 to enhance the generation of B10 cells, caution must be taken because IL-21 has also been demonstrated to promote T cell responses (29–33), and elevated levels of IL-21 have been implicated in driving autoimmune disease (34–36).

Our results differ somewhat from those published recently by Sheng et al. (19). in which they demonstrate a deficiency in the CD19^+^CD24^hi^CD38^hi^ Bregs to suppress type 1 T helper (Th1) cytokine production and had no effect of CD19^+^CD24^hi^CD38^hi^ Bregs in suppressing CD4 T cell proliferation. Variations in results could be attributed to differences in the approach to assessing functionality of Bregs and identification of Breg subsets. Sheng et al. characterized two populations of Bregs: Breg1 (CD19^+^CD1d^+^CD5^+^ B cells) and Breg2 (CD19^+^CD38^+^CD24^+^ B cells). By contrast, we identified a broader population of Bregs with the common property of IL-10 production. This was intentional, as IL-10-producing B cells overlap in phenotype with multiple proposed Breg subsets, including those selected by Sheng et al., and other phenotypes (21, 37). Currently, a known specific transcription factor or cell surface phenotype that identifies Bregs is undefined (21). We also found that 48 h of stimulation with LPS (not shown) or CpG optimized CD19^+^ IL-10-producing B cells (Figure 1). Sheng et al. compared Breg2 depletion versus no depletion to assess the production of Th1 cytokines. They demonstrate that depleting the Breg2 population in MG patients did not increase the frequency of IFN-γ- and TNF-α-producing CD4^+^ T cells, suggesting that the Breg2 subset in MG is defective in suppressing T cell function. In their approach, the inability to suppress could be attributed to other Breg subsets including B10 cells that are keeping the CD4 T cell response at bay even after Breg2 cells are depleted. Furthermore, our transwell experiment demonstrated that the majority of B10 cell suppressive activity on CD4^+^ T cells is likely through secreted IL-10.

Since the suppression of CD4 proliferation in the transwell condition did not match a mixed 1:1 culture of B cells and T cells, it is likely that other B cell-mediated immunosuppressive mechanisms are involved. One possibility is the suppression through direct or indirect cell-to-cell contact. Early Breg studies defining cell-to-cell contact as a mechanism of B cell-mediated immunosuppression stimulated isolated CD19^+^CD25^+^ B cells with autologous CD4 T cells (38, 39). Although the CD19^+^CD25^+^ B cells significantly suppressed the proliferative capacity of CD4 T cells, further phenotypic analysis revealed that this population was associated with high levels of IL-10 (39). Furthermore, this group also observed enhanced suppression by Bregs after stimulation of Bregs with CpG ODN-CD40L. These observations do not discard the role of IL-10 in their observed Breg suppression, since we include CpG ODN-CD40L to promote the generation of B10 cells. Furthermore, the Bregs can indirectly promote T cell suppression by promoting the expression of FOXP3 and CTLA-4 in Tregs (39).

Collectively, we demonstrate a defect in the quantity and quality of B10 cells in MG patients with moderate to severe disease. Unlike other B cell subsets in which the phenotype consists of a heterogenous population of Bregs and other B cells, B10 cells are a distinct population of Bregs with IL-10 as the common identifier. These results suggest that B10 cell frequencies may be a useful biomarker of disease severity, and therapeutics designed to restore B10 cell frequencies could hold promise as a treatment for this disease through restoration of self-tolerance.

## Author Contributions

JY and JG contributed to the conception and design of the study. MR was responsible for experimental procedures and data acquisition. JM, VJ, LH-W, KG, SR, and KB were involved in data acquisition. KW was involved in data interpretation. All authors contributed to drafting the manuscript and approve the final version. They also agree to the accuracy and integrity of the manuscript.

## Conflict of Interest Statement

The authors declare that the research was conducted in the absence of any commercial or financial relationships that could be construed as a potential conflict of interest.

## Abbreviations

AChR, acetylcholine receptor; Breg, regulatory B cell; LPS, lipopolysaccharide; MG, myasthenia gravis; MMT, manual muscle testing; PBMC, peripheral blood mononuclear cell; PMA, phorbal 12-myristate 13-acetate; Th1, type 1 T helper; TLR, toll-like receptor; Tregs, regulatory T cells.

## References

[1] Berrih-Aknin S Le Panse R. Myasthenia gravis: a comprehensive review of immune dysregulation and etiological mechanisms. J Autoimmun (2014) 52:90–100. 10.1016/j.jaut.2013.12.011 24389034

[2] Balandina A Lecart S Dartevelle P Saoudi A Berrih-Aknin S Functional defect of regulatory CD4(+)CD25+ T cells in the thymus of patients with autoimmune myasthenia gravis. Blood (2005) 105:735–41. 10.1182/blood-2003-11-3900 15454488 PMC1847365

[3] Sheng JR Li LC Ganesh BB Prabhakar BS Meriggioli MN. Regulatory T cells induced by GM-CSF suppress ongoing experimental myasthenia gravis. Clin Immunol (2008) 128:172–80. 10.1016/j.clim.2008.03.509 18502693 PMC2536633

[4] Thiruppathi M Rowin J Ganesh B Sheng JR Prabhakar BS Meriggioli MN Impaired regulatory function in circulating CD4(+)CD25(high)CD127(low/-) T cells in patients with myasthenia gravis. Clin Immunol (2012) 145:209–23. 10.1016/j.clim.2012.09.012 23110942 PMC3501560

[5] Yi JS Guidon A Sparks S Osborne R Juel VC Massey JM Characterization of CD4 and CD8 T cell responses in MuSK myasthenia gravis. J Autoimmun (2014) 52:130–8. 10.1016/j.jaut.2013.12.005 24378287 PMC4230445

[6] Mizoguchi A Mizoguchi E Smith RN Preffer FI Bhan AK. Suppressive role of B cells in chronic colitis of T cell receptor alpha mutant mice. J Exp Med (1997) 186:1749–56. 10.1084/jem.186.10.1749 9362534 PMC2199135

[7] Fillatreau S Sweenie CH McGeachy MJ Gray D Anderton SM. B cells regulate autoimmunity by provision of IL-10. Nat Immunol (2002) 3:944–50. 10.1038/ni833 12244307

[8] Mauri C Gray D Mushtaq N Londei M. Prevention of arthritis by interleukin 10-producing B cells. J Exp Med (2003) 197:489–501. 10.1084/jem.20021293 12591906 PMC2193864

[9] Matsushita T Yanaba K Bouaziz JD Fujimoto M Tedder TF. Regulatory B cells inhibit EAE initiation in mice while other B cells promote disease progression. J Clin Invest (2008) 118:3420–30. 10.1172/JCI36030 18802481 PMC2542851

[10] Watanabe R Ishiura N Nakashima H Kuwano Y Okochi H Tamaki K Regulatory B cells (B10 cells) have a suppressive role in murine lupus: CD19 and B10 cell deficiency exacerbates systemic autoimmunity. J Immunol (2010) 184:4801–9. 10.4049/jimmunol.0902385 20368271 PMC3734559

[11] Carter NA Rosser EC Mauri C. Interleukin-10 produced by B cells is crucial for the suppression of Th17/Th1 responses, induction of T regulatory type 1 cells and reduction of collagen-induced arthritis. Arthritis Res Ther (2012) 14:R32. 10.1186/ar3736 22315945 PMC3392827

[12] Le Huu D Matsushita T Jin G Hamaguchi Y Hasegawa M Takehara K Donor-derived regulatory B cells are important for suppression of murine sclerodermatous chronic graft-versus-host disease. Blood (2013) 121:3274–83. 10.1182/blood-2012-11-465658 23422748

[13] Duddy M Niino M Adatia F Hebert S Freedman M Atkins H Distinct effector cytokine profiles of memory and naive human B cell subsets and implication in multiple sclerosis. J Immunol (2007) 178:6092–9. 10.4049/jimmunol.178.10.6092 17475834

[14] Blair PA Norena LY Flores-Borja F Rawlings DJ Isenberg DA Ehrenstein MR CD19(+)CD24(hi)CD38(hi) B cells exhibit regulatory capacity in healthy individuals but are functionally impaired in systemic lupus erythematosus patients. Immunity (2010) 32:129–40. 10.1016/j.immuni.2009.11.009 20079667

[15] Newell KA Asare A Kirk AD Gisler TD Bourcier K Suthanthiran M Identification of a B cell signature associated with renal transplant tolerance in humans. J Clin Invest (2010) 120:1836–47. 10.1172/JCI39933 20501946 PMC2877933

[16] Pallier A Hillion S Danger R Giral M Racape M Degauque N Patients with drug-free long-term graft function display increased numbers of peripheral B cells with a memory and inhibitory phenotype. Kidney Int (2010) 78:503–13. 10.1038/ki.2010.162 20531452

[17] Sun F Ladha SS Yang L Liu Q Shi SX Su N Interleukin-10 producing-B cells and their association with responsiveness to rituximab in myasthenia gravis. Muscle Nerve (2014) 49:487–94. 10.1002/mus.23951 23868194

[18] Guptill JT Yi JS Sanders DB Guidon AC Juel VC Massey JM Characterization of B cells in muscle-specific kinase antibody myasthenia gravis. Neurol Neuroimmunol Neuroinflamm (2015) 2:e77. 10.1212/NXI.0000000000000077 25745635 PMC4345633

[19] Sheng JR Rezania K Soliven B. Impaired regulatory B cells in myasthenia gravis. J Neuroimmunol (2016) 297:38–45. 10.1016/j.jneuroim.2016.05.004 27397074

[20] Ding T Yan F Cao S Ren X. Regulatory B cell: new member of immunosuppressive cell club. Hum Immunol (2015) 76:615–21. 10.1016/j.humimm.2015.09.006 26385479

[21] Lykken JM Candando KM Tedder TF Regulatory B10 cell development and function. Int Immunol (2015) 27:471–7. 10.1093/intimm/dxv046 26254185 PMC4817073

[22] Yanaba K Bouaziz JD Haas KM Poe JC Fujimoto M Tedder TF A regulatory B cell subset with a unique CD1dhiCD5+ phenotype controls T cell-dependent inflammatory responses. Immunity (2008) 28:639–50. 10.1016/j.immuni.2008.03.017 18482568

[23] Iwata Y Matsushita T Horikawa M Dilillo DJ Yanaba K Venturi GM Characterization of a rare IL-10-competent B-cell subset in humans that parallels mouse regulatory B10 cells. Blood (2011) 117:530–41. 10.1182/blood-2010-07-294249 20962324 PMC3031478

[24] Jaretzki A III Barohn RJ Ernstoff RM Kaminski HJ Keesey JC Penn AS Myasthenia gravis: recommendations for clinical research standards. Task force of the medical scientific advisory board of the Myasthenia Gravis Foundation of America. Neurology (2000) 55:16–23. 10.1212/WNL.55.1.16 10891897

[25] Sanders DB Tucker-Lipscomb B Massey JM A simple manual muscle test for myasthenia gravis: validation and comparison with the QMG score. Ann N Y Acad Sci (2003) 998:440–4. 10.1196/annals.1254.057 14592912

[26] Yoshizaki A Miyagaki T Dilillo DJ Matsushita T Horikawa M Kountikov EI Regulatory B cells control T-cell autoimmunity through IL-21-dependent cognate interactions. Nature (2012) 491:264–8. 10.1038/nature11501 23064231 PMC3493692

[27] Wang RX Yu CR Dambuza IM Mahdi RM Dolinska MB Sergeev YV Interleukin-35 induces regulatory B cells that suppress autoimmune disease. Nat Med (2014) 20:633–41. 10.1038/nm.3554 24743305 PMC4048323

[28] Sheng JR Quan S Soliven B CD1d(hi)CD5+ B cells expanded by GM-CSF *in vivo* suppress experimental autoimmune myasthenia gravis. J Immunol (2014) 193:2669–77. 10.4049/jimmunol.1303397 25135828 PMC4167377

[29] Yi JS Du M Zajac AJ. A vital role for interleukin-21 in the control of a chronic viral infection. Science (2009) 324:1572–6. 10.1126/science.1175194 19443735 PMC2736049

[30] Iannello A Boulassel MR Samarani S Debbeche O Tremblay C Toma E Dynamics and consequences of IL-21 production in HIV-infected individuals: a longitudinal and cross-sectional study. J Immunol (2010) 184:114–26. 10.4049/jimmunol.0901967 19949086

[31] Yi JS Ingram JT Zajac AJ. IL-21 deficiency influences CD8 T cell quality and recall responses following an acute viral infection. J Immunol (2010) 185:4835–45. 10.4049/jimmunol.1001032 20844201 PMC2950881

[32] Chevalier MF Julg B Pyo A Flanders M Ranasinghe S Soghoian DZ HIV-1-specific interleukin-21+ CD4+ T cell responses contribute to durable viral control through the modulation of HIV-specific CD8+ T cell function. J Virol (2011) 85:733–41. 10.1128/JVI.02030-10 21047960 PMC3020027

[33] Spolski R Leonard WJ. Interleukin-21: a double-edged sword with therapeutic potential. Nat Rev Drug Discov (2014) 13:379–95. 10.1038/nrd4296 24751819

[34] Caruso R Botti E Sarra M Esposito M Stolfi C Diluvio L Involvement of interleukin-21 in the epidermal hyperplasia of psoriasis. Nat Med (2009) 15:1013–5. 10.1038/nm.1995 19684581

[35] Nakou M Papadimitraki ED Fanouriakis A Bertsias GK Choulaki C Goulidaki N Interleukin-21 is increased in active systemic lupus erythematosus patients and contributes to the generation of plasma B cells. Clin Exp Rheumatol (2013) 31:172–9. 23137515

[36] Deng XM Yan SX Wei W. IL-21 acts as a promising therapeutic target in systemic lupus erythematosus by regulating plasma cell differentiation. Cell Mol Immunol (2015) 12:31–9. 10.1038/cmi.2014.58 25088225 PMC4654374

[37] Barsotti NS Almeida RR Costa PR Barros MT Kalil J Kokron CM. IL-10-producing regulatory B cells are decreased in patients with common variable immunodeficiency. PLoS One (2016) 11:e0151761. 10.1371/journal.pone.0151761 26991898 PMC4798727

[38] Tretter T Venigalla RK Eckstein V Saffrich R Sertel S Ho AD Induction of CD4+ T-cell anergy and apoptosis by activated human B cells. Blood (2008) 112:4555–64. 10.1182/blood-2008-02-140087 18802006

[39] Kessel A Haj T Peri R Snir A Melamed D Sabo E Human CD19(+)CD25(high) B regulatory cells suppress proliferation of CD4(+) T cells and enhance Foxp3 and CTLA-4 expression in T-regulatory cells. Autoimmun Rev (2012) 11:670–7. 10.1016/j.autrev.2011.11.018 22155204

