# Supplementary material for: B10 Cell Frequencies and Suppressive Capacity in Myasthenia Gravis Are Associated with Disease Severity
Source: Front Neurol. 2017 Feb 10;8:34. doi: 10.3389/fneur.2017.00034 (PMC5301008; doi:10.3389/fneur.2017.00034)
Supplement: Supplementary file 1 [file table_1.docx]

**Supplementary Table 1. Clinical characteristics of AChR MG patients at the time of first study blood draw (N=64)**

| **Subj #** | **Age (Yr)** | **Race** | **MGFA Severity Class** | **MG-MMT** | **Thymectomy** | **MG Immunosuppressives** |
| --- | --- | --- | --- | --- | --- | --- |
| 1 | 86 | W | 3A | 38 | No | Pred 10mg/d |
| 2 | 47 | W | 1 | 1 | Yes | MMF 750mg/d |
| 3 | 59 | W | 1 | 12 | No | None |
| 4 | 82 | W | 1 | 2 | No | Pred 15mg/d |
| 5 | 74 | W | 2B | 15 | No | Pred 80mg/d |
| 6 | 66 | W | 2A | 3 | No | MMF 2000mg/d |
| 7 | 79 | W | 2A | 15 | Yes | Pred 15mg/d |
| 8 | 55 | W | 2A | 2 | Yes | RTX (12 months prior) |
| 9 | 62 | W | 3B | 19 | No | None |
| 10 | 64 | W | 1 | 3 | Yes | Pred 1.25 mg/d  MMF 2000mg/d |
| 11 | 61 | W | 1 | 11 | No | None |
| 12 | 50 | W | 1 | 2 | Yes | None |
| 13 | 56 | W | 1 | 7 | No | None |
| 14 | 71 | W | 2B | 2 | No | MMF 2500mg/d  Pred 2.5mg/d |
| 15 | 67 | W | 3B | 29 | No | None |
| 16 | 25 | A | 1 | 2 | No | None |
| 17 | 76 | W | 3B | 18 | No | None |
| 18 | 48 | W | 2B | 4 | No | None |
| 19 | 30 | W | 1 | 4 | No | None |
| 20 | 35 | W | 0 | 0 | Yes | None |
| 21 | 19 | B | 3B | 28 | No | TPE/IVIG^a^ |
| 22 | 74 | W | 2B | 5 | No | None |
| 23 | 72 | W | 2B | 6 | No | None |
| 24 | 69 | W | 3A | 18 | No | None |
| 25 | 66 | W | 0 | 0 | No | None |
| 26 | 69 | W | 3B | 4 | No | Pred 60mg/d |
| 27 | 65 | W | 4B | 16 |  | None |
| 28 | 56 | W | 2B | 7 | No | None |
| 29 | 61 | W | 2B | 6 | No | AZA 100mg/d |
| 30 | 71 | B | 2A | 14 | Yes | None^b^ |
| 31 | 68 | W | 1 | 3 | No | MMF 2000mg/d |
| 32 | 67 | W | 2B | 3 | Yes | AZA 100mg/d |
| 33 | 82 | W | 1 | 4 | No | Pred 3.75mg/d  AZA 150mg/d |
| 34 | 26 | B | 1 | 2 | No | Pred 10mg/d  AZA 200 mg/d |
| 35 | 57 | W | 1 | 7 | No | MMF 3000 mg/d |
| 36 | 60 | W | 0 | 0 | Yes | AZA 50 mg/d |
| 37 | 71 | W | 1 | 4 | No | None |
| 38 | 69 | W | 3B | 14 | No | None |
| 39 | 48 | W | 2B | 3 | No | AZA 350mg/d |
| 40 | 35 | B | 2A | 13 | Yes | None |
| 41 | 79 | W | 2A | 10 | No | None |
| 42 | 64 | W | 1 | 6 | No | Pred 20mg/d |
| 43 | 77 | W | 1 | 2 | No | Pred 2.5mg/d |
| 44 | 74 | W | 1 | 2 | No | Pred 2.5mg/d  MMF 2000mg/d |
| 45 | 64 | W | 2B | 3 | No | Pred 5mg/d  AZA 200mg/d |
| 46 | 53 | B | 1 | 1 | No | Pred 15mg/d  MMF 2500mg/d |
| 47 | 62 | W | 1 | 1 | No | MMF 2500mg/d |
| 48 | 77 | W | 1 | 1 | No | None |
| 49 | 28 | W | 1 | 2 | No | Pred 20mg/d |
| 50 | 26 | B | 2A | 11 | No | AZA 100mg/d |
| 51 | 32 | B | 3B | 24 | No | MMF 3000 mg/d |
| 52 | 54 | B | 2A | 10 | No | Pred 30mg/d |
| 53 | 51 | W | 2A | 12 | No | None |
| 54 | 68 | B | 3A | 35 | Yes | TPE^a^ |
| 55 | 59 | NA | 0 | 0 | No | MMF 500mg/d |
| 56 | 32 | A | 3A | 18 | No | None |
| 57 | 20 | B | 3A | 31 | No | None |
| 58 | 47 | B | 4B | 13 | No | None |
| 59 | 62 | W | 5 | 10 | No | Pred 40mg/d  TPE^a^ |
| 60 | 70 | B | 2B | 4 | No | None |
| 61 | 48 | B | 1 | 5 | Yes | Pred 2.5mg/d  AZA 100mg/d |
| 62 | 73 | W | 1 | 4 | No | Pred 10mg/d |
| 63 | 81 | W | 0 | 1 | No | Pred 7.5mg/d  MMF 2000mg/d |
| 64 | 78 | W | 0 | 0 | Yes | Pred 10mg/d |

Abbreviations: A=Asian; AZA=azathioprine; B=black; d=day; g=grams; I=improved; MG=Myasthenia Gravis; mg=milligrams; MGFA=Myasthenia Gravis Foundation of America; MMF=mycophenolate mofetil; MG-MMT=myasthenia gravis manual muscle testing score at time of blood draw; NA=Native American; Pred=prednisone; RTX=rituximab; TPE=therapeutic plasma exchange; W=white; Yr=years

^a^Within 90 days of blood draw

^b^cyclophosphamide, cisplatin and doxorubicin chemotherapy 90 days preceding blood draw
